# Selective enhancement of topologically induced interface states in a dielectric resonator chain

**DOI:** 10.1038/ncomms7710

**Published:** 2015-04-02

**Authors:** Charles Poli, Matthieu Bellec, Ulrich Kuhl, Fabrice Mortessagne, Henning Schomerus

**Affiliations:** 1 Department of Physics, Lancaster University, Lancaster LA1 4YB, UK; 2 Laboratoire de Physique de la Matière Condensée, CNRS UMR 7336, Université Nice Sophia Antipolis, 06100 Nice, France

## Abstract

The recent realization of topological phases in insulators and superconductors has advanced the search for robust quantum technologies. The prospect to implement the underlying topological features controllably has given incentive to explore optical platforms for analogous realizations. Here we realize a topologically induced defect state in a chain of dielectric microwave resonators and show that the functionality of the system can be enhanced by supplementing topological protection with non-hermitian symmetries that do not have an electronic counterpart. We draw on a characteristic topological feature of the defect state, namely, that it breaks a sublattice symmetry. This isolates the state from losses that respect parity-time symmetry, which enhances its visibility relative to all other states both in the frequency and in the time domain. This mode selection mechanism naturally carries over to a wide range of topological and parity-time symmetric optical platforms, including couplers, rectifiers and lasers.

## Abstract (web summary)

At interfaces between systems with topologically distinct band structure, robust symmetry protected states emerge. Here, Poli *et al.* control such states in a coupled dielectric resonator chain with parity-time symmetry and exploit their topological nature to protect them from absorptive losses.

## Graphical abstract

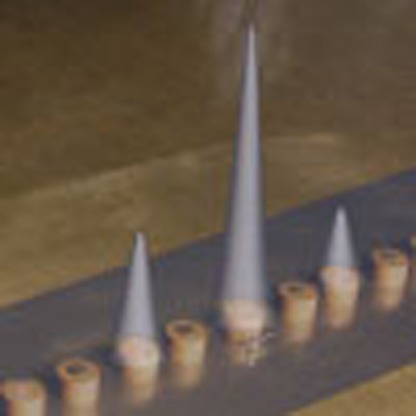

Topological photonics^1^ aims to implement robust optical modes by mirroring the interference and interaction effects that drive condensed matter into topologically protected phases^2^,^3^. A key element for the intended topological functionality are robust confined states that form at interfaces between regions with topologically distinct band structures. For electromagnetic waves, this can be realized in two-dimensions by breaking symmetries in analogy to the quantum Hall effect^4^,^5^,^6^ or the quantum spin Hall effect^7^,^8^,^9^, while in one-dimension one simply can employ lattice modulations^10^,^11^,^12^. Concerning the latter setting, a minimal one-dimensional model with a topological band structure is a chain of sites with alternating couplings (that is, a dimer chain). This model was originally introduced in an electronic context by Su, Schrieffer and Heeger (SSH)^13^ to describe fractionalized charges in polyacetylene, which appear in the presence of a dimerization defect. Photonic systems provide a versatile platform to realize analogies of this situation. The topological defect state has been observed in a quantum walk scenario^12^, while a dimer chain with a non-topological defect has been realised in a waveguide array^14^.

In absence of the defect, this dimer model has attracted independent attention because it provides a natural platform for gain-loss distributed systems displaying a so-called parity-time 
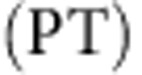
 symmetry^15^,^16^,^17^,^18^,^19^. The topological features of the PT-symmetric variant of the chain without a defect has been discussed in^20^,^21^, while two recent experiments have exploited spontaneous PT-symmetry breaking for mode selection in a laser^22^,^23^. This relies on a mechanism where two modes with real-frequencies coalesce and bifurcate in a strongly and weakly amplified mode^24^,^25^,^26^.

The intrinsic robustness of topologically induced states raises the general question whether they can be controlled and modified independently of the other states in the system. It is then natural to consider whether non-hermitian effects without an electronic analogue, such as embodied by the losses and gain in PT-symmetric systems, may be of any help in the photonic setting.

Here we demonstrate the selective control and enhancement of the topologically induced state in the SSH chain in a one-dimensional microwave setup. We draw on the passive variant of non-hermitian PT-symmetry^15^,^17^,^19^ and implement the chain in presence of the defect and localized absorptive losses by means of a set of identical coupled dielectric resonators placed in a microwave cavity^27^,^28^,^29^,^30^. The defect state explicitly breaks the PT symmetry^31^,^32^; this topologically induced anomaly further simplifies the mode competition. The state can then be isolated from losses affecting all other modes in the system, which enhances its visibility in the temporal evolution of a pulse, even in presence of structural disorder. As the explicit symmetry breaking is a general characteristic feature of topologically induced interface states, our results transfer to a wide range of settings. Besides its relevance for mode guiding and filtering, as well as rectifiers and couplers exploiting passive PT symmetry, this mechanism also lays the conceptual ground for selecting a topologically induced state in mode competition in active variants of the symmetry, tying it to the topical problem of gain–loss enabled lasing.

## Results

### Realization of dimer chains by coupled resonators

**Figure 1:**
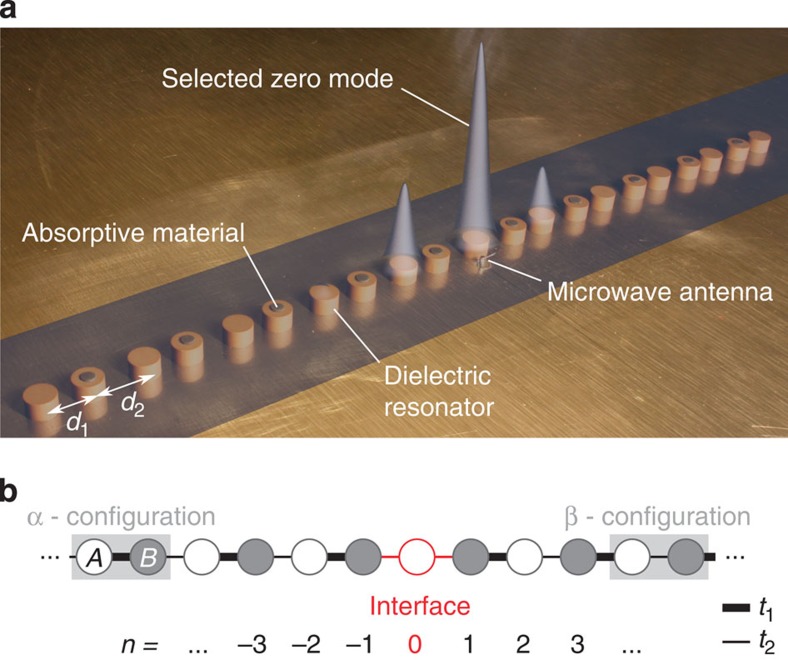
Realization of a topological defect in a microwave resonator chain. (**a**) Picture of the experimental microwave realization of the complex Su-Schrieffer-Heeger (SSH) chain. The lattice is composed of 21 identical coupled dielectric cylindrical resonators (5 mm height, 8 mm diameter and a refractive index of 6) sandwiched between two metallic plates (note that the top plate is not shown). To implement dimer chains, the resonators are separated by spacings *d*_1_ or *d*_2_ with *d*_1_<*d*_2_, that is, couplings *t*_1_>*t*_2_. A central dimerization defect is introduced by repeating the spacing *d*_2_. The defect creates an interface state at zero energy, a zero mode, whose visibility is enhanced by means of absorptive patches placed on one of the two sublattices. The resulting wavefunction intensity is superimposed onto the chain. (**b**) Schematic of the complex SSH chain, with *A* and *B* sublattices indicated in white and grey, respectively. The strong (weak) coupling strength is represented by a thick (thin) line. In our system, the couplings can be controlled by varying the resonator spacings. The topologically induced zero-mode appears at the interface (red) between α configuration (with strong intradimer coupling) and β configuration (with weak intradimer coupling).

Figure 1a depicts a chain of 21 microwave resonators with a central dimerization defect. We establish a one-dimensional tight-binding regime^27^, where the electromagnetic field is mostly confined within the resonators. For an isolated resonator, only a single mode is important in a broad spectral range around the bare frequency *ν*_b_=6.65 GHz. This mode spreads out evanescently, so that the coupling strength can be controlled by adjusting the separation distance between the resonators^27^. The resulting system can be described by the following tight-binding equations:


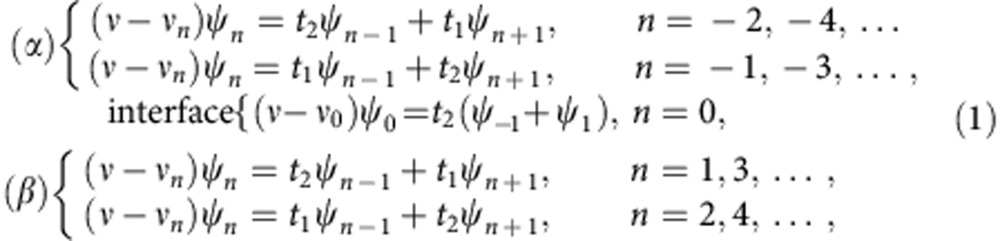


where *n* enumerates the resonators, with *n*=−10,−9,−8,...10 and *n*=0 for the central site (see Fig. 1b). The mode amplitude in the *n*th resonator is given by 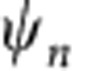, *t*_1_ and *t*_2_ denote the alternating nearest-neighbour coupling strengths, while *ν*_*n*_ is the resonance frequency of the *n*th isolated resonator. Without absorption, the resonance frequencies are equal to the bare frequency, *ν*_*n*_=*ν*_b_. Absorption is introduced on selected sites by depositing elastomer patches on top of the dielectric cylinders (see Fig. 1a). The losses give rise to a complex resonance frequency 
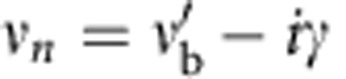
, and also shift the real part of the bare frequency to 
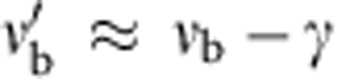
. In our experiments, *γ*≃40 MHz, while the separations *d*_1_=12 mm and *d*_2_=15 mm correspond to couplings *t*_1_=37.1 MHz and *t*_2_=14.8 MHz, respectively.

### Behaviour of defect-free chains

**Figure 2:**
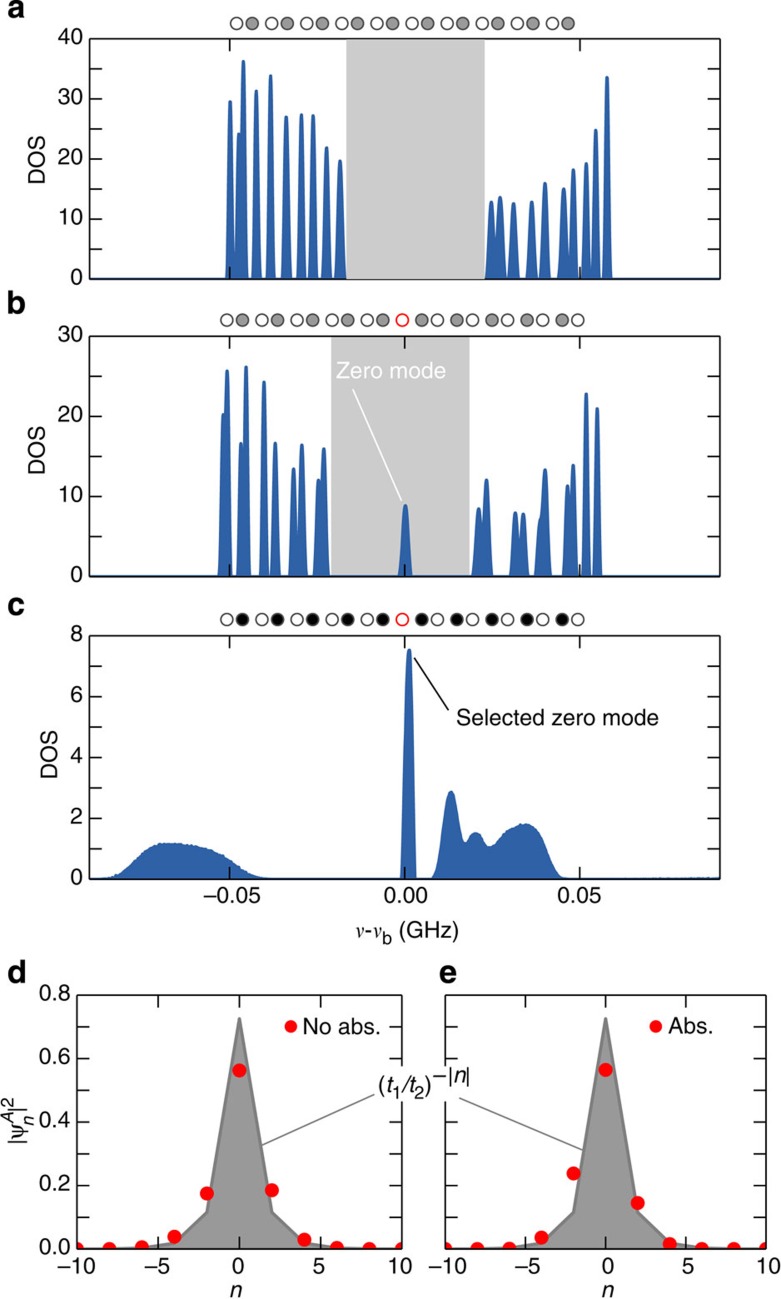
Density of states and zero-mode profiles with and without absorption. (**a**) Experimentally obtained density of states (DOS) for a defectless SSH chain (no interface, see inset above the main panel where open circles denote A sites and grey filled circles denote B sites) for separation distances *d*_1_=12 mm (coupling *t*_1_=37.1 MHz) and *d*_2_=15 mm (*t*_2_=14.8 MHz). The reference frequency is the bare frequency of an isolated resonator *ν*_b_=6.65 GHz. Two bands separated by a gap (grey zone) are observed. (**b**) DOS obtained for an SSH chain with a dimerization defect (inset, red circle). A zero-mode appears in the band gap. (**c**) DOS obtained for a complex SSH chain dimerization defect (inset, red circle) and absorption located on *B* sites (black filled circles in the inset). While all the extended modes experience losses, the zero-mode is preserved. (Note that the ordinate axes scales are different). (**d**) Experimental intensity profile of the zero-mode on the *A* sites (red dots), along with the theoretical intensity profile ∝(*t*_1_/*t*_2_)^−|*n*|^ (shaded in grey), for the chain without absorption. The intensity is zero on *B*-sites, and the total intensity is normalized to unity. (**e**) Intensity profile of the zero mode in presence of losses on the *B*-sites.

As a reference situation, we first consider a defectless SSH chain without absorption. As depicted in Fig. 2a, the density of states (DOS) is characterized by a band structure with two bands separated by a finite gap of size 2|*t*_1_–*t*_2_|=45 MHz (grey zone). The extended states occupy bands in the range *ν*_b_–*t*_1_–*t*_2_<*ν*<*ν*_b_–|*t*_1_–*t*_2_| and *ν*_b_+|*t*_1_–*t*_2_|<*ν*<*ν*_b_+*t*_1_+*t*_2_. The DOS is not affected when the values of the couplings *t*_1_ and *t*_2_ are interchanged. Nevertheless, a topological distinction between these two situations (called hereafter α and β configurations) can be captured by a winding number associated to the Bloch wave functions (see refs 31, 32, Supplementary Note 1 and Supplementary Figs 1 and 2). An interface between both configurations takes the form of a dimerization defect where two consecutive couplings are identical (red zone in Figs 1b and 2b). The topological distinctiveness of the two phases leads to the formation of an exponentially localized midgap state at *ν*=*ν*_b_. The corresponding wavefunction can be read off from equation (1), and takes the form 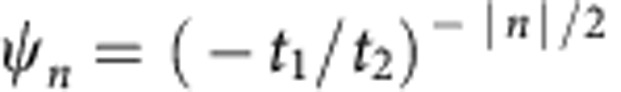 for even *n* and 
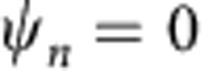
 for odd *n*. The midgap state is therefore confined to the sublattice with even index, which we will call the *A*-sublattice, while the sites with odd index are called the *B*-sublattice. The complementary state on the *B*-sublattice increases exponentially and is incompatible with the boundary conditions.

### Effects of dimerization defect

Figure 2b shows the DOS measured for a 21-resonator SSH chain with a central dimerization defect, still without absorption. Twenty-one modes are observed within the spectral range of interest. Of these, 20 modes are extended over the whole system. These modes group into two sets of 10 and correspond to the upper and the lower band of the infinite dimer chain. The bands remain separated by a 45 MHz gap. The topologically induced mode clearly sits the middle of the gap, at frequency *ν*_b_. We find that the intensity of the midgap state belonging to the *B*-sublattice is zero within experimental resolution, and thus confirm that the wavefunction is confined to the *A*-sublattice. The corresponding wavefunction intensity profile pertaining to the *A* sites is depicted in Fig. 2d (red dots). As expected, the intensity decays according to an exponential profile given by the theoretical result (shown in grey).

### Selective enhancement of topological states

We now set out to enhance the visibility of the topologically induced state. Our approach rests on the realization that the topological features of the system extend to a staggered configuration of losses, obtained by depositing absorptive material on all *B* sites^32^. Both in the α and in the β configuration, the tight-binding system then still possesses a passive PT-symmetry, given by a reflection 
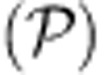
 at a point in the middle of a dimer, which maps the passive *A* sites onto the lossy *B* sites but leaves the couplings unchanged. This mapping corresponds to a time-reversal operation 
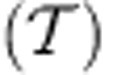
 up to a constant complex frequency shift *iγ*. As a consequence of this symmetry, all extended states are uniformly suppressed by the losses (their complex resonance frequencies all acquire the same imaginary part −*γ*/2). As it only lives on the *A*-sublattice, the topologically induced interface state manifestly breaks the PT-symmetry. In consequence, it does not have a complex–conjugate partner, and can be manipulated independently of all the other states in the system (which is not the case in situations where the PT-symmetry in the chain is only spontaneously broken^20^,^21^). Thus, the midgap state remains pinned at *ν*=*ν*_b_ and is unaffected by the losses (see Supplementary Note 1).

The spectral analysis presented in Fig. 2c shows that the extended modes shift downwards in frequency and become broadened, while the overall spectral weight in the resulting continuous bands is reduced. These features are consistent with the frequency dressing on the *B* sites, *ν*_b_→*ν*_b_–*γ–iγ*. In contrast, the peak in the density of states associated to the zero mode remains fixed at the bare frequency *ν*_b_, and its height and width is almost unchanged. As shown in Fig. 2e this mode remains well confined to the *A*-sublattice, and still displays an exponential intensity profile as one moves away from the defect site. Under the same conditions, non-topological defect states hybridize, thereby degrading their properties, see Supplementary Note 2 and Supplementary Figs 3 and 4.

**Figure 3:**
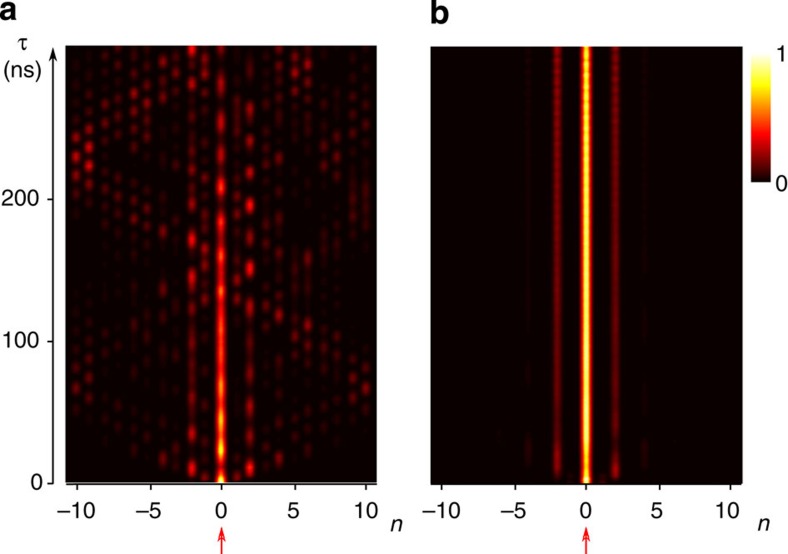
Selective enhancement of the zero mode. (**a**) Time evolution of the field intensity (normalized at each time step and colour coded by brightness; maximal intensity white, vanishing intensity black) in the SSH chain without absorption. The initial excitation is localized at the defect site at the centre of the system (indicated by the red arrow). All modes are participating in the propagation. (**b**) Time evolution of the field intensity in the complex SSH chain with absorption on *B*-sites. The zero mode is enhanced and dominates in the propagation.

The spectral analysis implies that the interface state is insensitive to the losses distributed on the *B* sites. It then should become the predominant mode during the time evolution. To illustrate this feature, Fig. 3 shows the time evolution for both non-absorptive (a) and absorptive (b) cases, corresponding to a pulse launched on the defect site. Without absorption, diffraction and interferences spoil the propagation of the interface state, which cannot be discerned after 250 ns. On the contrary, adding losses drastically enhances the visibility of the topologically induced mode, which then dominates the propagation without any degradation.

### Robustness against structural disorder

**Figure 4:**
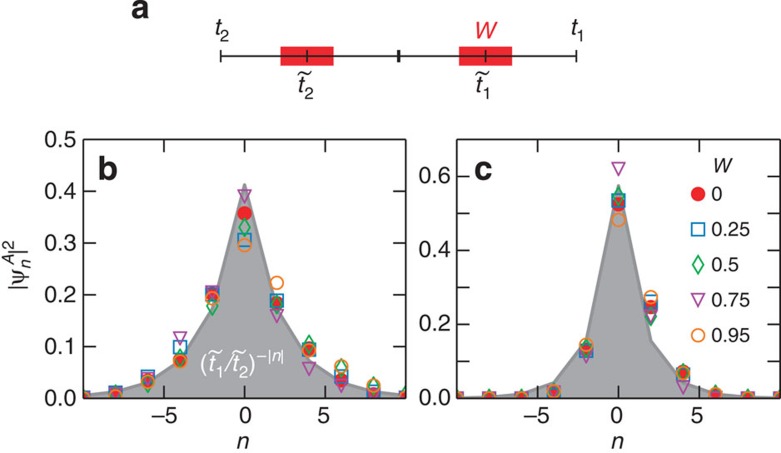
Robustness of the zero mode against structural disorder. (**a**) Schematic representation of the randomized coupling strengths *t*_1_, *t*_2_ used in the experiments. The disorder strength *W* (red bar) is chosen to preserve the topological structure of the chain (*t*_1_>*t*_2_). (**b**) Zero-mode intensity profiles (normalized to 1) on *A* sites for the disordered chain without losses for different values of *W*. The grey-shaded curve follows the exponential profile 
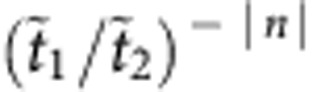
. (**c**) Intensity profiles in the presence of losses on the *B*-sites. The grey-shaded curve is obtained for an effective coupling ratio 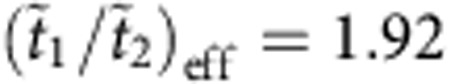. The topologically induced interface state is seen to be robust against the structural disorder, both with and without absorption.

To probe the topological protection property of the midgap state, we introduce structural disorder by randomly distributing the intersite separations. In the tight-binding description, this corresponds to a random modification of the coupling strengths. We preserve the dimer structure by defining the couplings as 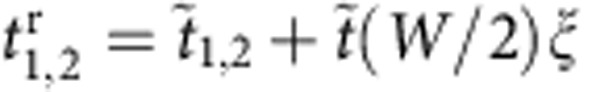, where 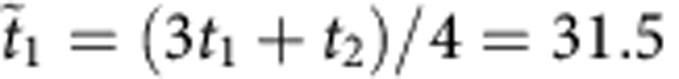 MHz, 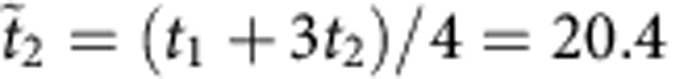 MHz and 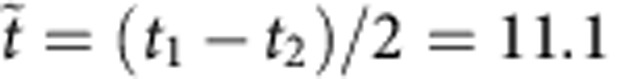 MHz. Here *W* is the disorder strength varying from 0 to 0.95 and *ξ* is a random number uniformly distributed in the interval [−1, 1] (see Fig. 4a). As shown in Fig. 4b, for different values of *W* the zero-mode intensities exhibit a very similar profile: an approximately exponential decay on the *A* sites and insignificant intensity on the *B* sites. Even though we do not perform any averaging over disorder realizations, the experimental profile is in remarkable agreement with the simple exponential 
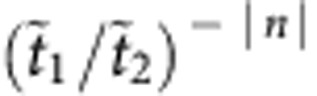
. This robustness in presence of disorder arises from the sublattice (chiral) symmetry and is further enhanced by the existence of a finite gap. Note that as 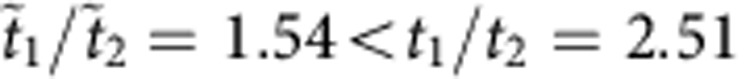 the present zero modes are more extended compared with the situation considered previously (Fig. 2d). When absorption is added on *B* sites, we observe in Fig. 4c that the robustness of the mode persists. Due to the resonance frequency shift of the lossy resonators, the couplings are now slightly changed to an effective coupling ratio 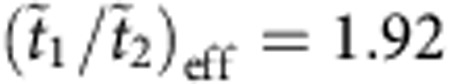, which is taken into account in the theoretical profile (grey-shaded curve).

## Discussion

The loss-induced selective enhancement of the topological interface state observed in this work exploits the unique structure of the zero-mode wavefunction, which is confined to a sublattice. Such sublattice-symmetry breaking is common in topologically induced states; for example, it also occurs for the 0th Landau level in the quantum Hall effect of massless relativistic particles^33^, as well as in inhomogeneously strained graphene^34^ and photonic analogues of deformed honeycomb lattices^35^,^36^. The combination of topological constraints and passive PT-symmetry therefore provides a generic concept which may be exploited in different settings. This also extends to atom–optical systems, where the defectless version of the passive SSH model has recently been realized in an optical lattice^37^,^38^, while the non-passive case has been discussed in theoretical work^39^. We note that it is also possible to selectively suppress the interface state, which can be achieved either by placing the losses on the other sublattice or by interchanging the couplings (topological phases) on both sides of the defect. The enhancement or suppression of the state can therefore be used to detect the relative size of two coupling strengths. It is also attractive to replace the losses by amplification, for example, in a layered structure of materials with different thickness, which could be used to realize a laser with a much simplified mode competition, or by nonlinear effects as occurring, for example, in chains of coupled quantum dot or quantum well exciton polaritons^40^.

## Methods

### Microwave realization of tight-binding systems

The experimental setup is designed to realize a microwave system that is well approximated by a nearest-neighbour tight-binding description^27^. The sites of the lattice are occupied by dielectric microwave resonators with a cylindrical shape (Temex-Ceramics, E2000 series: 5 mm height, 8 mm diameter and a refractive index of 6). The resonance frequency of an isolated resonator *ν*_b_ is around 6.65 GHz and corresponds to the on-site energy of atoms in a tight-binding model. The dielectric cylinders are coupled by the evanescent electromagnetic field, the corresponding coupling strength *t* between two resonators is well described by a tight-binding-like hopping term; *t* depends on the separation *d* between resonators. Via a reflection measurement, one has access, at each site, to the local density of states and to the wavefunction intensity associated to each eigenfrequency. The DOS is obtained by averaging the local density of states over all resonator positions. To obtain the time evolution of the pulse we measure the transmission between a source located at the interface site and a receiver successively placed at each site position. By performing a Fourier transform, one obtains the temporal evolution of a pulse initiating from the defect site and propagating into the SSH chain. In all these experiments, we face an intrinsic on-site disorder of ~0.15% in the values of *ν*_b_.

## Author contributions

C.P. and H.S. developed the concept and theory, M.B. and F.M. performed the experiments and analysed the data. All the authors participated in the discussions and in writing the paper.

## Additional information

**How to cite this article:** Poli, C. *et al.* Selective enhancement of topologically induced interface states in a dielectric resonator chain. *Nat. Commun.* 6:6710 doi: 10.1038/ncomms7710 (2015).

## Supplementary Material

Supplementary Information Supplementary Figures 1-4, Supplementary Notes 1-2 and Supplementary References
