# Supplementary material for: Selective enhancement of topologically induced interface states in a dielectric resonator chain
Source: Nat Commun. 2015 Apr 2;6:6710. doi: 10.1038/ncomms7710 (PMC4396359; doi:10.1038/ncomms7710)
Supplement: Supplementary Information — Supplementary Figures 1-4, Supplementary Notes 1-2 and Supplementary References [file ncomms7710-s1.pdf]

Supplementary information

I. SUPPLEMENTARY FIGURES

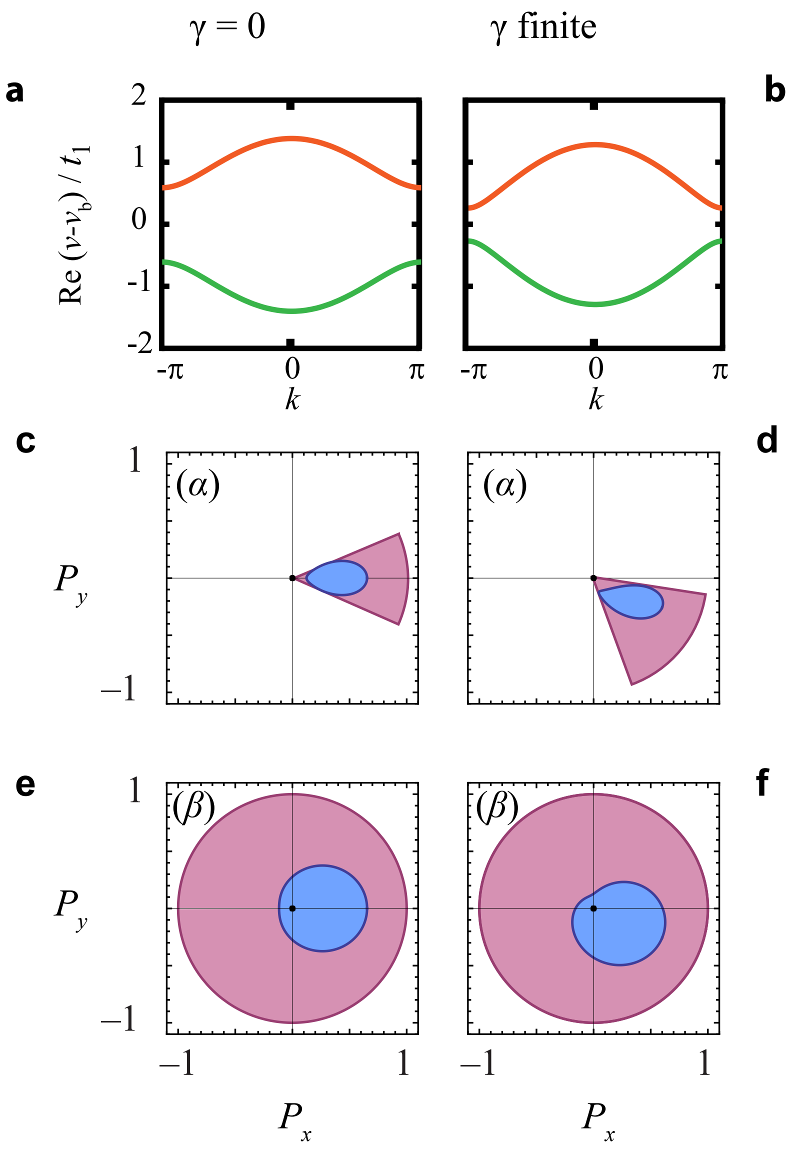

Supplementary Figure 1. **Band structure and topological characterization of the dimer chain without and with losses. a, b.** Theoretical dispersion relation of the tight-binding chain for couplings $t_a = t_1 = 37.1\,\mathrm{MHz}$, $t_b = t_2 = 14.8\,\mathrm{MHz}$ (α configuration), as realized in the experiment. In **a** the system is loss-free while in **b** the losses are set to $\gamma \simeq 40\,\mathrm{MHz}$, shifting the bare frequency $\nu_\mathrm{b} \to \nu_\mathrm{b} - i\gamma$ on the B sites. The losses reduce the band gap and uniformly shift the extended states into the complex plane, with $\mathrm{Im}\,\nu(k) = -\gamma/2$. For clarity we neglect the frequency-dressing shift of the real part. The dispersion in the β configuration is identical. **c-f.** The red outer curves show the trace of the polarization vector in the xy plane. In the α configuration (**c,d**), this curve describes a libration while in the β configuration (**e,f**) it describes a rotation. The blue curve shows the function $g(k) = (\nu(k) - \nu_\mathrm{b})f(k) \propto P_x + iP_y$ in appropriately scaled units in the complex $P_x + iP_y$ plane. In the α configuration, the origin lies outside the region encircled by $g(k)$, while in the β configuration the origin is enclosed.

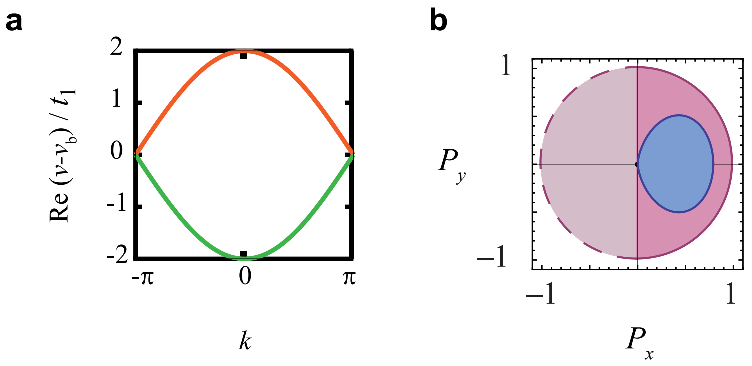

Supplementary Figure 2. **Band structure and topological characterization of the monomer chain. a.** Theoretical dispersion relation of the tight-binding monomer chain for $t_a = t_b = t_1$, in absence of any losses. **b.** As the gap closes, the blue curve showing $g(k) \propto P_x + iP_y$ passes through the origin, and the system is at the switching point between the α and β configurations.

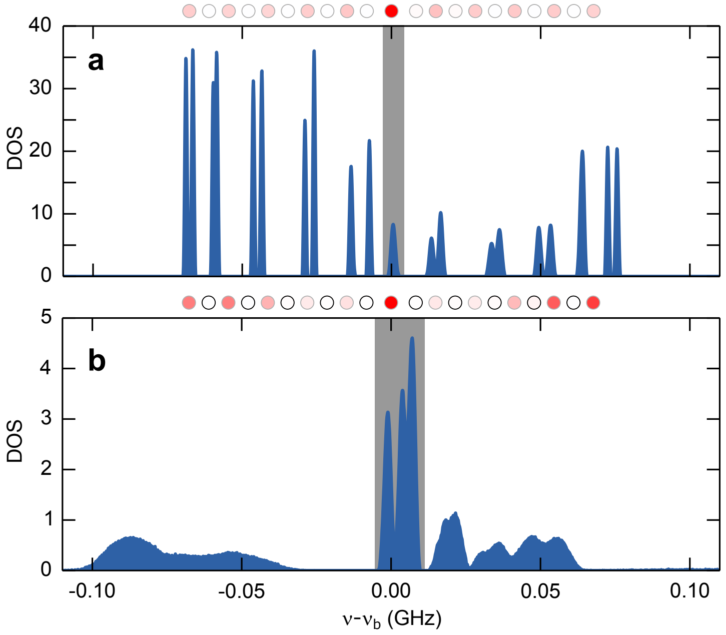

Supplementary Figure 3. **Density of states and zero-mode wavefunction intensities in a monomer chain with a non-topological defect. a.** Chain of 21 regularly spaced disks ($d_1 = 12\,\mathrm{mm}$) with a central embedded spacing defect ($d_2 = 15\,\mathrm{mm}$). This induces a zero mode (corresponding to the gray zone) with an enhanced intensity on the defect site, and a non-decaying tail on the A-sublattice (see inset above the panel, which encodes the intensity in red). **b.** Same as **a** but with losses on the B sites (indicated in the inset by the black contours). The zero-mode hybridizes, resulting in three broadened peaks of reduced height and an enhanced fluctuating intensity in the tails.

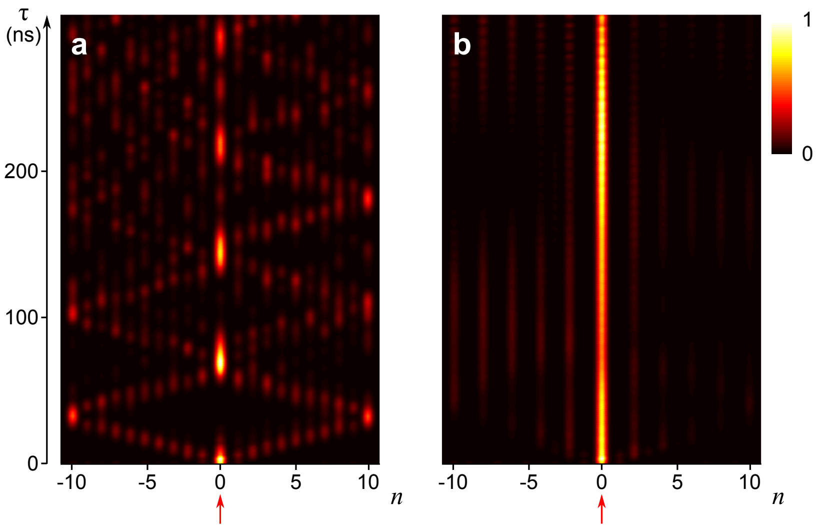

Supplementary Figure 4. **Temporal evolution of a pulse in the monomer chains with a non-topological defect. a.** Pulse propagation in absence of losses, with the intensity encoded by brightness (maximal intensity white, vanishing intensity black). **b.** Same as **a**, but with losses on B sites. The intensity has been normalized at every time step. The red arrows indicate the site position of the initial excitation.

II. SUPPLEMENTARY NOTES

Supplementary Note 1. Theoretical background

Topological characterization of the dimer chain without losses.— The topological features of the band structure of the dimer chain arise from a winding number associated to the Bloch wavefunctions [1, 2]. Let us first consider the defect-free system in absence of losses. The tight-binding model is then of the form

$$(\nu - \nu_b)\psi_n = t_{n+1}\psi_{n+1} + t_n\psi_{n-1} \tag{S.1}$$

with $t_n = t_a$ when n is odd and $t_n = t_b$ when n is even. For $0 < t_a < t_b$ the system is in the α configuration while for $0 < t_b < t_a$ it is in the β configuration. To distinguish A and B sites we write $\psi_{2n} = \phi_n^A$ and $\psi_{2n+1} = \phi_n^B$. Bloch wavefunctions can then be written as $\phi_n^{A,B} = \phi_{A,B}(k)\exp(ikn)$ with $\phi_A = 2^{-1/2}$, $\phi_B = \pm 2^{-1/2}(f(k)/|f(k)|)$, where $f(k) = t_a + t_b \exp(ik)$, k being the wavenumber. The corresponding eigenfrequency is $\nu(k) = \nu_b \pm |f(k)|$, where the plus sign gives the upper band and the minus sign gives the lower band. These bands are shown in the left panel of Supplementary Fig. 1a.

The vector $(\phi^A(k), \phi^B(k))^T$ can be interpreted as a pseudospin, and thus can be characterized by a polarization vector $\mathbf{P}(k) = (\langle\sigma_x\rangle, \langle\sigma_y\rangle, \langle\sigma_z\rangle)$ composed out of the expectation values of the Pauli matrices. This vector obeys $|\mathbf{P}(k)| = 1$. For the Bloch wavefunctions encountered here $\langle\sigma_z\rangle = 0$, so that $\mathbf{P}(k)$ is confined to the xy plane, with the direction given by $P_x + iP_y \propto (\nu(k) - \nu_b)f(k) \equiv g(k)$. As shown in the left panels of Supplementary Fig. 1b, in the α configuration this vector performs a libration (winding number 0), while in the β configuration it performs a rotation (winding number 1). This difference in the winding number is a topological feature since one cannot smoothly transform between both situations without closing the gap, violating the normalization of $\mathbf{P}(k)$ or leaving the xy plane. In the figure, the blue curve traces out the function $g(k)$ in the complex $P_x + iP_y$ plane. In the α configuration the curve does not encircle the origin, but in the β configuration it does.

When these two topologically distinct configurations are connected as described by Eq. (1) in the main text, a midgap state appears which is localized at the interface. For a general discussion of topological interface states see [3, 4]. In the present case, this state can be inferred directly by setting $\nu = \nu_b$, $\phi_n^B = 0$, giving the exponential decay $\phi_n^A = (-t_1/t_2)^{-|n|}$ on both sides of the interface [i.e., in terms of the amplitudes in Eq. (S.1) $\psi_n = (-t_1/t_2)^{-|n|/2}$ for even n]. The exponential decay corresponds to an imaginary wave number, at which $f(k) = 0$. The broken sublattice symmetry results in a polarization vector $\mathbf{P}(k) = (0, 0, 1)$, pointing out of the xy plane.

Topological characterization in presence of losses.— Up to a finite threshold $|\gamma| < \gamma_0 = 2|t_a - t_b|$, these qualitative features survive the introduction of losses on the B sites, corresponding to a shift of the onsite frequency on these sites to $\nu_n = \nu_b - i\gamma$ [5]. The eigenfrequencies for the Bloch wavefunctions shift to $\nu(k) = \nu_b - i\gamma/2 \pm \sqrt{|f(k)|^2 - \gamma^2/4}$, meaning that all extended states display the same amount of loss-induced decay. The real part of the dispersion relation is shown in the right panel of Supplementary Fig. 1a. The pseudospin vector is given by $\phi_A = 2^{-1/2}$, $\phi_B = 2^{-1/2} f(k)/(\nu(k) - \nu_b)$, and the polarization vector is still confined to the xy plane, as shown in the right panels of Supplementary Fig. 1b. The characterization of the band structure in terms of winding numbers is thus preserved. In the presence of an interface, the existence of the topologically induced state can again be inferred directly from the tight-binding equations (1) in the main text, giving rise to the same frequency and wavefunction profile as in absence of losses.

For $|\gamma| > \gamma_0$ the interface state persists, but the gap closes, the losses of the extended states become state-dependent, and their spectral weight overlaps with the interface state. Furthermore, the pseudospin vector moves out of the plane, which signifies the breakdown of the topological characterization of the system.

Supplementary Note 2. Non-topologically induced defect state

In order to further explore the separate roles of topological protection and spatially distributed losses it is useful to consider a modified set-up in which a defect state is induced via a conventional *non-topological* mechanism. This is achieved by steering the system to the transition point between the α and β configurations, which corresponds to a monomer chain with constant site spacing. The gap then closes already in absence of the losses, as the states of the two bands become degenerate at $k = \pm\pi$ (see Supplementary Fig. 2a). These bands can be interpreted as two branches of a single band in the unfolded Brillouin zone of the monomer chain. Out of the degenerate states at $k = \pm\pi$ one can then form superpositions that are confined either to the A or to the B-sublattice, with $\mathbf{P}(k) = (0, 0, \pm 1)$ just as for the interface state but now achieved for real k. Thus, the topological characterization of the system breaks down (see Supplementary Fig. 2b).

In our experiments, we set the uniform spacing to $d_1 = 12$ mm (coupling strength $t_1 = 37.1$ MHz) and maintain the embedded spacing defect at the position of the central resonator ($d_2 = 15$ mm, i.e. $t_2 = 14.8$ MHz). The system then still displays a defect mode at the bare frequency ν_b, but instead within a band gap this mode now lies in the

middle of the unfolded band. This is verified by the experimental spectral analysis depicted in Supplementary Fig. 3a, where the position of the state in the frequency spectrum is highlighted in gray. The corresponding wavefunction intensity (inset) shows that the defect mode is enhanced at the defect, but now possesses a non-decaying tail on the A-sublattice. This concurs with the prediction from the tight-binding model, according to which the tail is given by $|\psi_n|^2 = (t_2/t_1)^2|\psi_0|^2$ ($n \neq 0$ even).

It is interesting to note that in absence of any disorder we still find zero intensity on the B-sublattice, as for the topologically induced defect state. The persistence of the sublattice-polarization follows from the chiral symmetry of tight-binding model with a bipartite lattice structure [6, 7]. In the one-dimensional tight-binding chain this symmetry is realized trivially because A sites only couple to B sites and vice versa. However, as this feature depends on a symmetry it is far less robust than in the case of the topologically induced mode. For example, we find that a small amount of disorder now suffices to induce hybridization with other extended states in the system. In particular, hybridization already occurs when losses are introduced onto the B sites. As shown in Supplementary Fig. 3b, in the density of states several broadened peaks of reduced height then overlap in the region around the bare frequency. The associated spatial intensity distribution (inset) is still large at the defect site, but the weight in the tails increases, especially close to the edges of the system. In the time domain (see Supplementary Figs. 4a and b), the pulse propagation profile again stabilizes when absorption is added, but the intensity on the defect site fluctuates and an oscillating intensity remains clearly visible in the tails. Thus, in absence of topological protection the defect state is not isolated against the losses, making the propagation less robust than for the topologically induced state studied in the paper.

III. SUPPLEMENTARY REFERENCES

[1] Ryu, S. & Hatsugai, Y. Topological origin of zero-energy edge states in particle-hole symmetric systems. *Phys. Rev. Lett.* **89**, 077002 (2002).

[2] Delplace, P., Ullmo, D. & Montambaux, G. Zak phase and the existence of edge states in graphene. *Phys. Rev. B* **84**, 195452 (2011).

[3] Hasan, M. Z. & Kane, C. L. Topological insulators. *Rev. Mod. Phys.* **82**, 3045–3067 (2010).

[4] Qi, X.-L. & Zhang, S.-C. Topological insulators and superconductors. *Rev. Mod. Phys.* **83**, 1057–1110 (2011).

[5] Schomerus, H. Topologically protected midgap states in complex photonic lattices. *Opt. Lett.* **38**, 1912–1914 (2013).

[6] Lieb, E. H. Two theorems on the Hubbard model. *Phys. Rev. Lett.* **62**, 1201–1204 (1989).

[7] Brouwer, P. W., Racine, E., Furusaki, A., Hatsugai, Y., Morita, Y. & Mudry, C. Zero modes in the random hopping model. *Phys. Rev. B* **66**, 014204 (2002).
